# Impact of discontinuation of lactation consultant program on early infant feeding in Manitoba

**DOI:** 10.1186/s13006-025-00737-1

**Published:** 2025-05-26

**Authors:** Amy Hui, Jennifer M. Yamamoto, Roxana Dragan, Vanessa Poliquin, Patricia Birk, Katherine Kearns, Elizabeth Decaire, Vivian Omarr, Chukwudumebi Onyiuke, Kira Friesen, Christina Raimondi, Brandy Wicklow, Carol Dyck, Garry X. Shen

**Affiliations:** 1 Department of Internal Medicine, University of Manitoba, 835-715 McDermot Ave, Winnipeg, MB R3E 3P4 Canada; 2 Department of Community Health Sciences, Manitoba Center of Health Policy, University of Manitoba, Winnipeg, Canada; 3 Children Hospital Research Institute of Manitoba, Department of Pediatrics and Child Health, University of Manitoba, Winnipeg, Canada; 4 Department of Obstetrics, Gynecology and Reproductive Sciences, University of Manitoba, Winnipeg, Canada; 5 Department of Family Medicine, University of Manitoba, University of Manitoba, Winnipeg, Canada; 6 First Nations Health and Social Secretariat of Manitoba, Winnipeg, Canada; 7 Four Arrows Regional Health Authority, Winnipeg, Canada; 8 Health Sciences Centre (Winnipeg), Winnipeg, Canada; 9 Winnipeg Breastfeeding Centre, University of ManitobaWinnipeg Breastfeeding Centre, Winnipeg, Canada; 10 Youville Community Health Centre, Winnipeg, Canada

**Keywords:** Breastfeeding, Formula feeding, Lactation consultant program, Indigenous health

## Abstract

**Background:**

Lactation Consultants (LC) at Health Sciences Centre (HSC) and St Boniface General Hospitals (SBGH) supported the lactation of 2/3 of Manitoba newborns since 1994. The LC program in HSC was discontinued in 2018. Its impact on infant feeding in the province remains unclear.

**Methods:**

To assess the influence of the LC program cessation on the feeding of newborns in postpartum wards via a retrospective administrative database cohort.

**Results:**

A total of 126,285 infants were delivered in all Manitoban hospitals during 2014–2021 [First Nations (FN): 21%, all others: 79%, urban: 55%, rural: 41% and remote: 4%]. The rates of breastfeeding were lower and formula feeding were higher in FN and all other newborns after the program cessation (2018–2021) compared to that during 2014–2017 (*p* < 0.01). The intensity of the changes in infant feeding among FN or remote-living newborns during 2018–2021 were 2–threefold greater than that among all others or urban/rural-living newborns delivered in HSC (*p* < 0.01). In contrast, infant feeding status stayed stable for those delivered at SBGH where the LC program did not withdraw. The cessation of LC program decreased adjusted odds ratio (aOR) for exclusive breastfeeding in FN infants (aOR 0.93, 95% CI: 0.88–0.98) and urban-living infants (aOR 0.96, 95% CI: 0.94–0.98), but not in all others, rural- or remote-living infants. Increased odds for formula feeding was detected in FN and all other infants living in various regions in the province during 2018–2021 compared to that during 2014–2017 (*p* < 0.05).

**Conclusion:**

The findings suggest that the discontinuation of LC program decreased breastfeeding and increased formula feeding, and the unfavorable changes in infant feeding was most profound among FN and remote-living infants.

## Introduction

Breast milk has been recommended to all mothers by the World Health Organization (WHO) as the ideal food for infants. Breastfeeding provides short- and long-term health benefits to mothers and their babies [1]. Exclusive breastfeeding for ≥ 6 months offers the greatest advantages for both mothers and children [2]. Previous study indicated that a delayed onset of lactation is likely associated with shorter breastfeeding duration [3].

Breastfeeding could be difficult for some mothers due to health conditions of mothers or infants as well as socioeconomic factors, such as family support, environment and prenatal education. They need to acquire essential knowledge, skills for lactation and make decision to start feeding within a short period after experiencing intense physical and psychological stress during labor [4]. Perinatal education on breastfeeding after hospital admission helps mothers to build up motivation and preparation for breastfeeding. In addition, in-person support around labor plays a critical role in breastfeeding initiation, exclusive breastfeeding and reduced the risk of infant overweight and obesity in high income countries [5].

International board certified lactation consultants (IBCLCs) have been integrated into routine clinical practice of many hospitals in high-income countries since the middle of the 1980's [6]. IBCLCs in wards not only educate pregnant women on the benefits of breastfeeding but also provide personal supports to women with technical, physical, or psychological difficulties to initiate breastfeeding by themselves [7]. Previous studies demonstrated that IBCLCs effectively increased the rate of breastfeeding, particularly exclusive breastfeeding and breastfeeding initiation [8–12].

Manitoba has the highest percentage of First Nations (FN), which is the largest Indigenous group, in all provinces in Canada. FN women in Manitoba had lower rates of breastfeeding initiation compared to all other women [13], which may result from historical, geographical factors and socioeconomic barriers [14].

Manitoba had > 15,000 live-births annually between 2014 and 2021. Women’s hospital in Health Sciences Center (HSC) and St Boniface General Hospital (SBGH) are only two hospitals with obstetric facilities in Winnipeg. They provide labor service for 2/3 of all deliveries of pregnant women from urban, rural and remote areas in the province. In 1994, lactation consultant (LC) program was initiated at HSC and later in SBGH. IBCLCs in the LC program effectively supported mothers for breastfeeding initiation in the province until the end of 2017. The LC program at HSC, the largest hospital in the province, was discontinued on January 1, 2018 due to funding cuts in the provincial healthcare system. The influence of the cessation of the LC program in HSC on breastfeeding in the province remains unclear.

To assess the influence of the withdrawal of the LC program on the feeding status of infants delivered at HSC, a database was created to track the feeding of infants delivered in all hospitals in Manitoba between 2014 and 2021 finance years. This database will also enable us to assess the association between the cessation of the LC program and infant feeding of FN and all other infants residing in various regions of the province.

## Methods

### Databases

A retrospective database study was conducted using administrative databases linked with all living births delivered in hospitals in the province during the study period. The Manitoba Centre for Health Policy (MCHP) at the University of Manitoba maintains the data repository, which contains de-identified administrative databases for all residents of Manitoba enrolled under the universal healthcare plan. These databases include physician claims, hospital discharge abstracts, vital statistics, and pharmaceutical prescriptions linked using a scrambled personal health information number. Health data were collected and transferred to MCHP by health record technicians at Manitoba Health, who were unaware of the project's design. The reliability of the databases was previously validated [15]. To verify FN status in the study, linkages were made with the Indian Registry System database.

### Ethics approvals

The study received approvals from the Health Research Ethics Board (HREB) at the University of Manitoba, the Health Information Research Governance Committee in First Nations Health and Social Secretariat of Manitoba (FNHSSM), the provincial Health Research Privacy Committee and Shared Health Approval Committee for Privacy. Additional permission was obtained for use of FN identifier in the Status Verification System in the Repository from the following approval bodies: the Health Information Research Governance Committee of FNHSSM, the Department of Aboriginal and Northern Affairs Canada and the Indian Registry System. Participant consent was not required for the database study by HREB due to anonymity of participants’ information.

### Study population and timeframe

This study included all pregnancies of women and births of newborns residing in Manitoba, with deliveries in all hospitals with obstetric facilities between April 1, 2014 and March 31, 2022 (annual birth rate of each year in MCHP covers all deliveries from April 1 of index year to March 31 of next year). Exclusion criteria were: i) non-pregnant; ii) babies who died or did not have health insurance coverage within 3 months after delivery; iii) mothers with less than 1 year of health insurance coverage before the first day of gestation; iv) infants delivered outside of province or not within the study time frame.

### Outcomes and definitions

#### Breastfeeding initiation

The information about breastfeeding initiation before hospital discharge (usually < 48 h postpartum) was obtained from newborn feeding records in hospital abstracts stored in the Repository. This information included exclusive breastfeeding, mixed feeding, and formula feeding [13]. The definitions for these feeding types were:

- *Exclusive breastfeeding.* Newborns received only breast milk from their mothers before hospital discharge.
- *Mixed feeding.* Newborns were fed with breast milk and formula before hospital discharge.
- *Formula feeding. *Newborns were fed with formula only before hospital discharge.

### Confounding factors

#### Ethnicity

The FN status of pregnant women and their babies was self-reported by mothers and verified through National Registry System database with 99% of reliability as described [16]. Pregnant women without FN status and their babies were categorized into all other ethnic group women and offspring.

#### Residence regions

The residential address and zip codes provided by the mothers on hospital records were used to determine their locations, which were then categorized into urban, rural and remote regions within the province. Rural areas include all territory lying outside population centers beside remote regions [17]. Remote regions in Manitoba are defined as regions without all-weather road access, and require more than a four-hour drive from a major rural hospital, or have rail or fly-in access only [18].

#### Maternal age

Ages of mothers at deliveries were collected from hospital records.

### Statistical methods

Mean of percentages, adjusted odds ratio and 95% confidence of intervals of outcomes were assessed. The Chi-square test was used to assess the statistical difference of qualitative data. Crude ratios were assessed using saturated Poisson model. Odds ratios, 95% confidence intervals, and covariate adjustment were analyzed using generalized estimation equation regression model [19]. A significance level of *p* < 0.05 was set for statistical difference. All analyses were performed using SAS version 9.4 software.

## Results

A total of 126,285 live-births were delivered during 2014–2021 in all hospitals with obstetric facilities in Manitoba. Among them, 63,844 infants were delivered between 2014 and 2017 before the discontinuation of the LC program at HSC, and 62,441 infants were born after the cessation of the LC program between 2018 and 2021 (Table 1). Comparable distribution of FN and all other (ethnic groups) infants were delivered before and after the program cessation. Of the total infants, 55% resided in urban regions, 41% in rural regions and 4% in remote regions during both periods. For infants living in urban, rural or remote regions, 12%, 24% or 99% were FN descendants (Table 2).

**Table 1:** Annual total live-births in various regions in Manitoba between 2014 and 2021

	Manitoba	Urban regions	Rural regions	Remote regions
2014	15,893	8,684	6,525	684
2015	16,164	8,887	6,582	695
2016	15,650	8,679	6,328	643
2017	16,137	8,972	6,512	653
2018	15,942	8,803	6,516	623
2019	15,993	8,715	6,605	673
2020	15,356	8,336	6,386	643
2021	15,150	8,261	6,320	569

**Table 2:** First Nations (FN) and non-FN live births in Manitoba province between 2014 and 2021

	Manitoba (*n* = 126,085)	Urban regions (*n* = 698,337)	Rural regions (*n* = 51,774)	Remote regions (*n* = 5,174)
First Nations (%)	25,989 (21%)	8,196 (12%)	12,665 (24%)	5,128 (99%)
Non-First Nations (%)	100,296 (79%)	61,141 (87%)	39,109 (76%)	46 (1%)

**Fig. 1:**
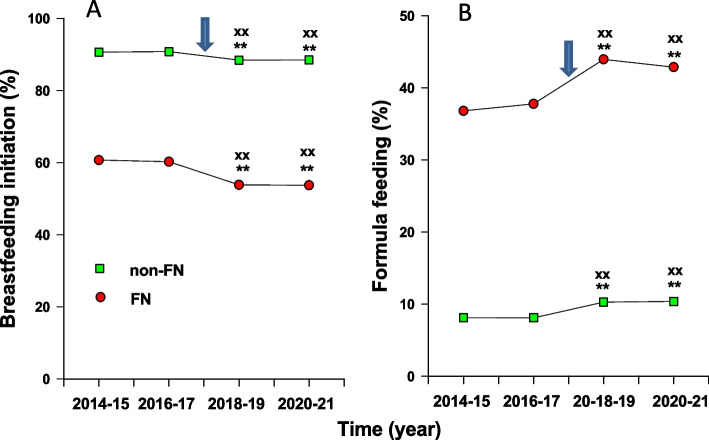
Influence of the discontinuation of lactation consultant (LC) program in Health Science Center (HSC) in Winnipeg on breastfeeding initiation for First Nations (FN) versus all other infants (non-FN) in Manitoba. **A** Breastfeeding initiation before hospital discharge including exclusive breastfeeding and mixed feeding (breast milk + formula) to FN (circles) and all other infants (squares) in Manitoba for periods of every two years between 2014 and 2021. **B** Formula feeding to FN or all other infants in Manitoba between 2014 and 2021. Arrows: time of the discontinuation of the LC program in HSC. **: *p* < 0.01 versus 2014–2015; ++ : *p* < 0.01 versus 2016–2017

During 2014–2017, 60.5% of FN infants and 90.7% of non-FN infants were breastfed, including exclusive breastfeeding or mixed feeding (breast milk plus formula), in postpartum wards before hospital discharge. The rates of breastfeeding during 2018–2021 to FN and non-FN infants after the cessation of the LC program were significantly lower than that during 2014–2017 (*p* < 0.01). The reduction of breastfeeding rates in non-FN infants during 2018–2021 compared to that during 2014–2017 was 2.2%, but the decrease of breastfeeding in FN infants during 2018–2021 compared to that during 2014–2017 was 6.7% (*p* < 0.01 versus all others, Fig. 1A, Table 3).

**Table 3:** Breastfeeding initiation in living births of different ethnic groups and residential locations before (2014–2017) and after discontinuation of lactation consultant program (2018–2021)

Period	First Nations	Non-First Nations	Urban	Rural	Remote
2014–17 case (%)	8,054 (60.5)	50,521 (90.7)	30,887 (87.7)	21,766 (83.9)	1,244 (46.5)
2018–21 case ()	6,806 (53.8)^**^	49,775 (88.5)^**^	28,804 (84.4)^**^	21,051 (81.5)^**^	988 (39.5)^**^
(2014–17)-(2018–21) case (%)	1,358 (6.7)	746 (2.2)^++^	1,235 (3.3)^xx^	715 (2.4)^xx^	256 (7.0)

^**^p < 0.01 versus 2014–17

^++^*p* < 0.01 versus FN group

^xx^*p* < 0.01 versus remote group

During 2014–2017, 8.1% of non-FN infants were fed with formula before hospital discharge, while 37.3% of FN infants received formula only during that period. The rate of formula feeding for both FN and all other infants during 2018–2021 were significantly higher than that during 2014–2017 (*p* < 0.01). The increase in formula feeding for all other infants during 2018–2021 versus 2014–2017 was 2.2%, but the increase in formula feeding in FN infants during 2018 versus 2014–2017 was 6.2% (*p* < 0.01, Fig. 1B, Table 4).

**Table 4:** Formula feeding in living births in in living births of different ethnic groups and residential locations before (2014–2017) and after discontinuation of lactation consultant program (2018–2021)

Period	First Nations	Non-First Nations	Urban	Rural	Remote
2014–17 case (%)	4,969 (37.3)	4,096 (8.1)	4,028 (11.4)	3,660 (14.1)	1,377 (51.4)
2018–21 case (5)	5,508 (43.5)^**^	5,144 (10.3)^**^	5,043(14.8)^**^	4,164 (16.1)^**^	1,445 (57.8)^**^
(2018–21)-(2014–17) case (%)	539 (6.2)	1,048 (2.2)^++^	1,015 (3.4)^xx^	504 (2.0)^xx^	68 (6.4)

^**^p < 0.01 versus 2014–17

^++^*p* < 0.01 versus FN group

^xx^*p* < 0.01 versus remote group

**Fig. 2:**
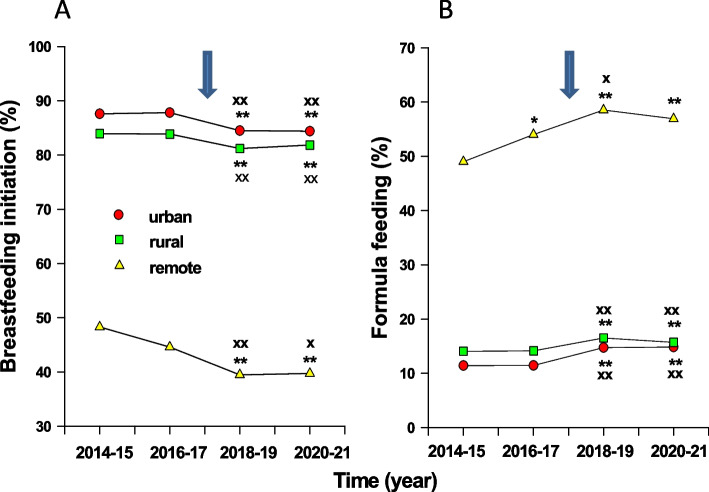
Influence of the cessation of LC program in HSC on breastfeeding initiation for babies living in various regions in Manitoba. **A** Breastfeeding initiation before hospital discharge to babies living in urban (Squares), rural (triangles) or remote regions (circles) in Manitoba for periods of every two years between 2014 and 2021. **B** Formula feeding to babies living various regions in Manitoba between 2014 and 2021. Arrows: time of the withdrawal of the LC program. **: *p* < 0.01 versus 2014–2015; +++ : *p* < 0.05 or 0.01 versus 2016–2017

Between 2014 and 2017, 87.7% and 83.9% of infants in urban and rural regions breastfed in hospital, but only 46.5% of infants living in remote regions, were breastfed in hospitals. The rates of breastfeeding in urban, rural and remote regions were significantly lower during 2018–2021 than that during 2014–2017 (*p* < 0.01, Fig. 2A). Decreases in breastfeeding in urban and rural regions between 2018–2021 and 2014–2017 periods were 3.3% and 2.4%, but the decrease of breastfeeding in remote regions was 7.0% (*p* < 0.01 versus urban or rural) (Table 3).

Between 2014 and 2017, 11.4% and 14.1% of infants in urban and rural regions, but 51.4% of infants living in remote regions, received formula only in hospitals. Figure 2B shows that the rates of formula feeding of infants living in urban, rural or remote regions during the period after the discontinuation of the LC program were significantly higher than that before the program withdrawal (*p* < 0.01). The increase of formula feeding in remote-living infants during 2018–2021 versus 2014–2017 was 6.4%, which was significantly higher than that in urban (3.4%) or rural regions (2.0%, *p* < 0.01 versus remote, Table 4).

**Fig. 3:**
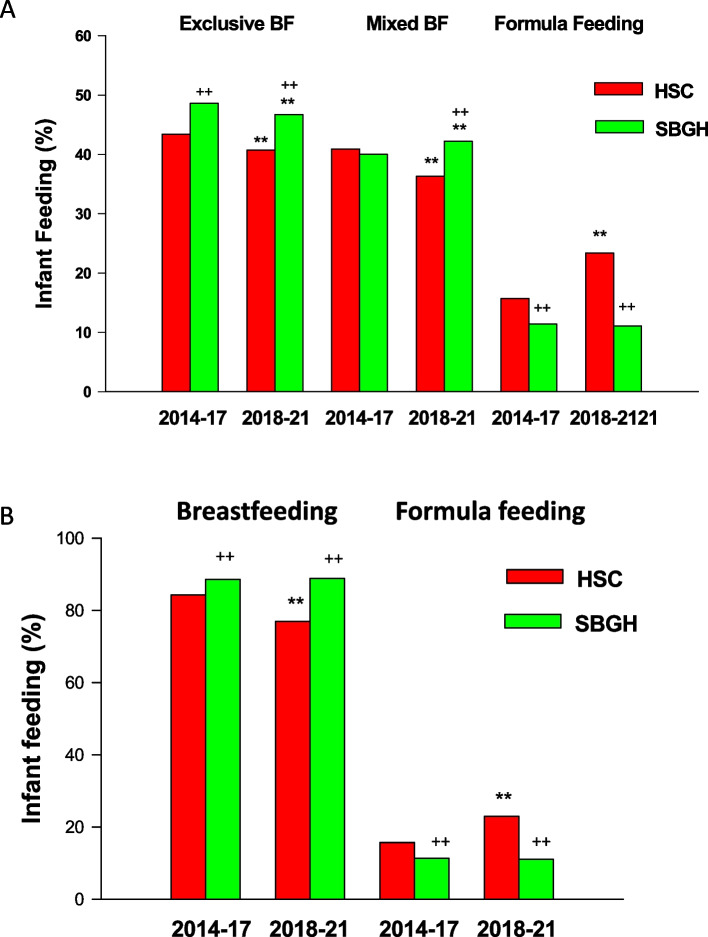
Influence of LC program cessation in HSC on breastfeeding and formula feeding in infants delivered in HSC compared to that in St Boniface General Hospitals (SBGH) without LC program cessation between 2014 and 2021. **A** exclusive breastfeeding, mixed feeding and formula feeding. **B** breastfeeding initiation and formula feeding. BF: breastfeeding. Red: infants delivered in HSC. Green: infants delivered in SBGH. **: *p* < 0.01 versus 2014–2017; ++ : *p* < 0.01 versus HSC

The rates of exclusive breastfeeding at SBGH during 2014–2017 and 2018–2021 periods were higher than that to infants delivered at HSC (p < 0.01). The rates of exclusive breastfeeding to infants delivered at HSC and SBGH during 2018–2021 were significantly lower than that to infants delivered during 2014–2017 (*p *< 0.01). After the cessation of the LC program at HSC, the rate of mixed feeding to infants delivered at HSC were significantly lower than that before the program cessation (*p* < 0.01), but the rate of mixed feeding to infants delivered at SBGH during 2018–2021 was significantly higher than that during 2014–2017 (*p* < 0.01). In addition, the rate of formula feeding to infants at HSC after the program cessation (2018–2021) was 52% higher versus during 2014–2017 (*p* < 0.01), but the rates of formula feeding to infants at SBGH between the two periods was not significantly different (Fig. 3A, Table 5). The rates of breastfeeding (exclusive breasting or mixed feeding) to infants delivered at HSC, but not those delivered at SBGH, were decreased during 2018–2021 versus that during 2014–2017 (*p* < 0.01, Fig. 3B).

**Table 5:** Infant feed in Health Science Center (HSC) versus St Boniface General Hospital (SBGH) during 2014–2017 compared to 2018–2021

Hospital	Period	Infants	Exclusive breastfeeding	Mixed feeding	Formula feeding
HSC	2014–2017	20,959	9,099 (43.4%)	8,578 (40.9%)	3,282 (15.7%)
HSC	2018–2021	21,715	8,829 (40.7%)^**^	7,890 (36.3%)^**^	4,996 (23.0%)^**^
SBGH	2014–2017	21,547	10,462 (48.6%)^++^	8,626 (40.2%)	2,459 (11.4%)^++^
SBGH	2018–2021	19,712	9,210 (46.7%)**^, ++^	8,919 (42.2%)^**, ++^	2,183 (11.1%)^++^

^**^p < 0.01 versus 2014–2017

^++^*p* < 0.01 versus HSC

**Fig. 4:**
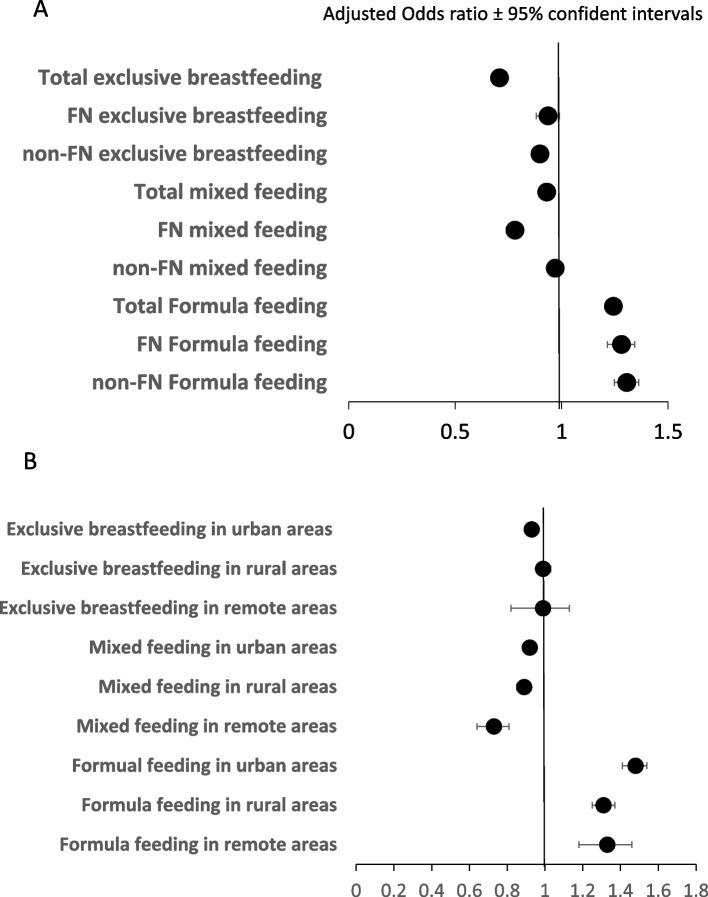
Influence of the discontinuation of LC program in HSC on adjusted odds on the types of feeding for FN and all other infants living in various regions in Manitoba. **A** adjusted odds ratio (aOR) and 95% confidence intervals (CI) for exclusive breastfeeding, mixed feeding and formula feeding prior to hospital discharge to total, FN or all other infants (non-FN) living in Manitoba after the withdrawal of LC program (2018–2021) versus that before the program suspension (2014–2017). **B** aOR and 95% CI for exclusive breastfeeding, mixed feeding and formula feeding before hospital discharge to infants living in various regions in Manitoba after the withdrawal of LC program (2018–2021) versus that before the program suspension (2014–2017)

The forest plot in Fig. 4 demonstrated that the adjusted odds ratios (aOR) and 95% confidence intervals (CI) for exclusive breastfeeding, mixed feeding, and formula feeding to FN and non-FN infants living in urban, rural or remote regions in Manitoba during 2018–2021 versus that during 2014–2017 after adjustments with maternal age and location. The results demonstrate that the withdrawal of LC program at HSC significantly reduced aOR for exclusive breastfeeding in total infants (aOR 0.95, 95% CI: 0.93–0.97), FN infants (aOR 0.93, 95% CI 0.88–0.98), and all other infants (aOR 0.96, 95% CI: 0.94–0.98) (*p* < 0.05). Besides, the LC program cessation significantly reduced the adjusted odds of mixed feeding and increased that of formula feeding in total, FN and all other infants (*p* < 0.05, Fig. 4A). After adjustment with age and ethnicity, the cessation of LC program reduced the odds for exclusive breastfeeding in urban-living infants (aOR 0.94, 95% CI 0.91–0.96, *p* < 0.05), but not that in rural (aOR 0.96, 95% CI: 0.95–1.02) or remote-living infants (aOR 0.99, 95% CI: 0.85–1.16). In addition, the program cessation reduced mixed feeding, and increased formula feeding to infants resided in all regions in the province (*p* < 0.05, Fig. 4B).

## Discussion

The findings of the present study demonstrated that the discontinuation of the LC program at one of major hospitals in the province, HSC, was associated with significantly lower rates of breastfeeding and higher rates of formula feeding to FN and all other infants across the province compared to those before the program cessation. The extents of decreases in breastfeeding and increases in formula feeding after the program cessation in FN and remote resided infants were 2–3-times greater than that in all other ethnic groups, urban and rural-living infants in Manitoba. The reduction in breastfeeding was only detected in infants delivered at HSC during LC program cessation, but not those delivered at SGBH, where the LC program remained operational during almost whole period between 2018 and 2021.

LC program is an important component of the perinatal breastfeeding support team. IBCLCs have specialized skills to educate and support pregnant women or new mothers to initiate and continue breastfeeding, and they have protected time, which allows them to offer holistic care for lactation support. Although many general ward nurses have knowledge and capacity to promote breastfeeding, they do not always have sufficient time to support complex breastfeeding needs in wards. The finding of the association between the LC program cessation and infant feeding in newborns delivered in HSC, but not in those delivered in SBGH, further confirms a causative role of the program cessation in the changes of infant feeding in affected newborns.

The results of the present study are consistent with the previous reports from other countries that demonstrated an LC program helps to increase breastfeeding [8–12, 20–24]. The findings of the present study demonstrated that the discontinuation of LC program in one major public hospital for four years resulted in the substantial reduction of breastfeeding and increase of formula feeding in newborns living in the province compared to historical record. The changes in the types of infant feeding potentially increase the risk of future diabetes in affected children [13]. The precise influence of the LC program cessation on long-term health of affected mothers and offspring in the province remains to be assessed in subsequent study.

The impact of the LC program on infant feeding may be affected by maternal age, ethnic group or residential location. The discontinuation of the LC program reduced the odds for exclusive breastfeeding in FN and all other infants. After the adjustment of maternal age and ethnic group, the reduction in the odds for exclusive breastfeeding was only detected in urban-living infants, but not in rural or remote-living infants. The findings suggest that without a significant change in exclusive breastfeeding to infants in rural or remote regions was partially depending on age or ethnic group. Younger pregnancy ages and high proportion of FN pregnant women in remote or rural regions possibly contributed to lower rates of exclusive breastfeeding to infants living in those regions. The absence of statistically significant change in exclusive breastfeeding in remote regions was likely due to relatively smaller sample size.

Human milk feeding reduces the risk of diabetes and cardiovascular diseases in mothers and offspring [13, 25, 26]. The results of the present study demonstrated that FN or remote living families and those living remote regions in Manitoba had more profound impact on infant feeding after the LC program cessation compared to that in non-FN, urban or rural living families. Breastfeeding is the traditional way of feeding babies in all populations including FN people. The lower rates of breastfeeding to FN or remote living infants may result from follows: i) many FN or remote living families lack of maternal teaching on breastfeeding due to cross-generational negative impact of colonialism and residential schools [14]; ii) higher unemployment rate and lower income in the majority of FN or remote living families limit parents to hire private IBCLCs or doulas to support breastfeeding; iii) FN and remote living pregnant women have additional barriers for prenatal education, such as housing, geographic factors and lack of nursing space in public facilities, which may contribute to lower breastfeeding and higher formula feeding for infants in FN or remote living families [27]; iv) most Northern remote communities in the province lack of high-speed internet, which hampered self e-education on breastfeeding by pregnant women in those regions [28]. The withdrawal of LC program in the largest public hospital in the province further limited opportunity of breastfeeding support to FN or remote living pregnant women, which resulted in more profound impact on infant feeding to their children and potentially aggravate health inequity in Indigenous people with socioeconomic disadvantages. The results of the present study emphasizes the urgent needs to reduce barriers in health system and general infrastructure for breastfeeding education to FN and remote living populations for improving the health of those women and children.

COVID-19 lowered breastfeeding rates in a study in Southern California [29]. The results of the present study demonstrated that significant reductions in breastfeeding in different ethnic groups and geographic regions after the program cessation in the province in 2018, which was before the start of COVID-19. The findings in the present study suggest that the cessation of LC program in a public hospital was likely an independent negative factor for infant breastfeeding. Although the present study did not compare the rates of breastfeeding before and after COVID-19, the results of another recent study by our group demonstrated that COVID-19 (March, 2020-Decemeber 2021) significantly decreased the rates of exclusive breastfeeding in FN and non-FN infants, and increased that of formula feeding in FN infants compared to a control period (March, 2018- March 2020) in Manitoba [30].

Limitations of the present study include follows: i) infant feeding information in the present study was based on hospital records collected before hospital discharge. Therefore, information of infants feeding for those delivered in homes and their breastfeeding duration were not included in the present study; ii) the administrative database in Manitoba does not include information on maternal body weights, the frequency of LC visits, prenatal education, or ethic groups beside FN, which were known to be relevant to breastfeeding initiation or duration [21, 22, 28, 31, 32].

The findings of the present study demonstrated that the LC program cessation in a major hospital was associated with significant decreases in breastfeeding and increases in formula feeding to newborns, which was more profound in FN or remote-living infants compared non-FN, urban or rural living infants. The LC program in HSC in Winnipeg was reactivated in February, 2024 after the results of the present study shared with health administrators and relevant healthcare team in the province. The authors intend to share this experience and findings with health administrators and healthcare workers in other regions or countries to make careful retention decision on existing LC program in public hospitals.

## Data Availability

Data will be available to share based on request to Dr. Garry X. Shen (garry.shen@umanitoba.ca).

## Abbreviations

aOR: Adjusted odds ratio
CI: Confidence intervals
FN: First Nations
FNHSSM: First Nations Health and Social Secretariat of Manitoba
HREB: Health Research Ethics Board
HSC: Health Sciences Centre (Winnipeg)
IBCLC: International Board Certified-Lactation Consultants
LC: Lactation consultant
MCHP: Manitoba Centre for Health Policy
SBGH: St Boniface General Hospital
